# Deep Learning-Based Segmentation of Peach Diseases Using Convolutional Neural Network

**DOI:** 10.3389/fpls.2022.876357

**Published:** 2022-05-25

**Authors:** Na Yao, Fuchuan Ni, Minghao Wu, Haiyan Wang, Guoliang Li, Wing-Kin Sung

**Affiliations:** ^1^College of Informatics, Huazhong Agricultural University, Wuhan, China; ^2^Hubei Engineering Technology Research Center of Agricultural Big Data, Wuhan, China; ^3^College of Information Engineering, Tarim University, Alaer, China; ^4^Department of Computer Science, National University of Singapore, Singapore, Singapore

**Keywords:** segmentation, location, peach diseases, focal loss, Mask R-CNN

## Abstract

Peach diseases seriously affect peach yield and people’s health. The precise identification of peach diseases and the segmentation of the diseased areas can provide the basis for disease control and treatment. However, the complex background and imbalanced samples bring certain challenges to the segmentation and recognition of lesion area, and the hard samples and imbalance samples can lead to a decline in classification of foreground class and background class. In this paper we applied deep network models (Mask R-CNN and Mask Scoring R-CNN) for segmentation and recognition of peach diseases. Mask R-CNN and Mask Scoring R-CNN are classic instance segmentation models. Using instance segmentation model can obtain the disease names, disease location and disease segmentation, and the foreground area is the basic feature for next segmentation. Focal Loss can solve the problems caused by difficult samples and imbalance samples, and was used for this dataset to improve segmentation accuracy. Experimental results show that Mask Scoring R-CNN with Focal Loss function can improve recognition rate and segmentation accuracy comparing to Mask Scoring R-CNN with CE loss or comparing to Mask R-CNN. When ResNet50 is used as the backbone network based on Mask R-CNN, the segmentation accuracy of segm_mAP_50 increased from 0.236 to 0.254. When ResNetx101 is used as the backbone network, the segmentation accuracy of segm_mAP_50 increased from 0.452 to 0.463. In summary, this paper used Focal Loss on Mask R-CNN and Mask Scoring R-CNN to generate better mAP of segmentation and output more detailed information about peach diseases.

## Introduction

Peach is an important and popular fruit, and its production is severely affected by peach diseases. The common peach diseases are brown rot, anthracnose, scab, bacterial shot hole, gummosis, powdery mildew, and leaf curl. The diseases reduce the yield of peach and cause harm to human health. Thus, it is important to find rapid and accurate methods to identify peach diseases and further locate and segment the areas of the lesion in earlier stages.

Currently, a few studies have been conducted on plant disease classification and on locating and segmenting areas of the lesion. There are three approaches. The first approach uses traditional image processing methods or deep learning methods to segment disease or pest areas initially. This preliminary segmentation is the intermediate step for feature extraction, which is the basic step for classification or location in the next step. Yang et al. (2018) used the Prewitt operator and the Canny operator for edge segmentation of single-headed pests based on the high contrast between the pest target and the background in the binary image, and then classified two types of pests by SVM, with the average recognition accuracy rate of 93.5%. Jin and Qian (2020) used fine-tune FCN to separate the diseased areas of green vegetables from the farmland images and then recognized the area by identifying the markers placed at a fixed distance on the ground, which can realize the location of the diseased area. The second approach focuses on classifying and identifying the diseases and further locating the lesion areas. Lu et al. (2017) used VGG-FCN-VD16 and VGG-FCN-S to classify the diseases and locate lesion areas, achieving the mean recognition accuracies of 97.95 and 95.12%, respectively. The third approach uses deep learning methods directly to segment the lesion site. Lin et al. (2019) used U-Net Ronneberger et al. (2015) network to segment cucumber leaves with powdery mildew and improved the segmentation effect by improving the loss function, thus achieving an average pixel accuracy of 96.08%, intersection over union of 72.11%, and dice accuracy of 83.45% on 20 test samples. Dai (2020) proposed a multi-scale fusion U-Net network to segment rice diseases. The first approach of segmentation is usually used for extracting preliminary features, such as the approximate location of the target. The second approach can provide disease classification and location based on the object detection task. The third approach can segment the lesion areas based on the semantic segmentation task. This study used deep learning methods to achieve classification, localization, and segmentation of peach diseases by instance segmentation task.

In deep learning methods, segmentation is initially carried out using FCN Long et al. (2015) network, and then other improved networks, such as DeconvNet Noh et al. (2016) and SegNet, are applied Badrinarayanan et al. (2017) Other networks for segmentation, such as DeepLab Chen et al. (2014) and PSPNet Zhao et al. (2017), are also available. The above-mentioned methods are based on semantic segmentation tasks. The FAST R-CNN (Girshick, 2015) approach can classify, identify, and locate targets, while Mask R-CNN (He et al., 2017) can not only classify and locate targets, but can also perform instance segmentation based on this information. At present, Mask R-CNN has been used for blade segmentation (Zhong et al., 2020), robot item recognition (Shi et al., 2019), pig inventory (Hu et al., 2020), and other applications. Some of the improved methods based on Mask R-CNN are Cascade R-CNN (Cai and Vasconcelos, 2019) and Deformable Convolutional Networks (Dai et al., 2017; Zhu et al., 2019). HRNet (Sun et al., 2019a,b) was also proposed for segmentation tasks. Mask Scoring R-CNN (Huang et al., 2019) adds a branch network on the basis of Mask R-CNN to train and regress mask scores.

This study focuses on identifying and locating major peach diseases and segmenting lesion areas using deep learning methods. The peach disease image dataset was collected from peach orchards by Prof. Luo’s team, College of Plant Science and Technology, HZAU, which included seven categories of peach disease images. The seven categories are as follows: (1) brown rot fungi infecting fruits and leaves, (2) anthracnose fungi infecting fruits and leaves, (3) scab fungus infecting fruits, branches, and leaves, (4) shot hole bacterium infecting fruits, branches, and leaves, (5) gummosis fungi infecting branches, (6) powdery mildew fungus infecting fruits and leaves, and (7) leaf curl fungus infecting leaves. These diseases cause damage to different parts of the peach plant. For example, the brown rot disease mainly infects the fruits, causing the fruit to rot, and also affects the leaves leading to the dryness of leaves. Gummosis mainly affects the branches, leading to tree weakness, decreased fruit quality, and ultimately causing the death of branches and trees. As the seven diseases were extensively studied in the laboratory, laboratory personnel were familiar with the characteristics of the diseases. For example, a certain disease mainly infects fruits, while some infect leaves and branches in particular. Therefore, the disease images were mainly obtained from the infected fruits. Each disease is further divided into early, middle, and end stages based on the severity of the disease. Finally, the total number of disease categories totals 21. The project comprises a team of experts on fruit disease prevention and control posts in the National Peach Industry Technology System, which can further ensure the accuracy of its classification. For similar diseases and diseases that are easy to be confused, accurate conclusions can be drawn through tissue isolation of pathogenic bacteria or direct monospore isolation, pathogen morphology observation, and molecular biological identification. The samples were collected by two methods. The first approach included collecting pictures of existing resources in the laboratory or obtaining some pictures from other experts through cooperation in the Peach System, and the second method included taking a large number of pictures indoors or in orchards.

For identifying disease, locating and segmenting lesion areas, two deep neural networks (Mask R-CNN and Mask Scoring R-CNN) are used to classify 21 diseases of peach trees and segment the lesions to obtain more detailed information about the lesions. To overcome the problem due to imbalance of samples and hard samples, by improving the loss function with focal loss (Lin et al., 2017) of Mask R-CNN and Mask Scoring R-CNN, the segmentation effect can be improved.

The remaining manuscript is organized as follows. Section 2 introduces “Materials and Methods.” Section 3 presents the “Results and related Discussion.” Finally, Section 4 presents our “Conclusion.”

## Materials and Methods

### Peach Disease Image Dataset and Image Annotation

**FIGURE 1:**
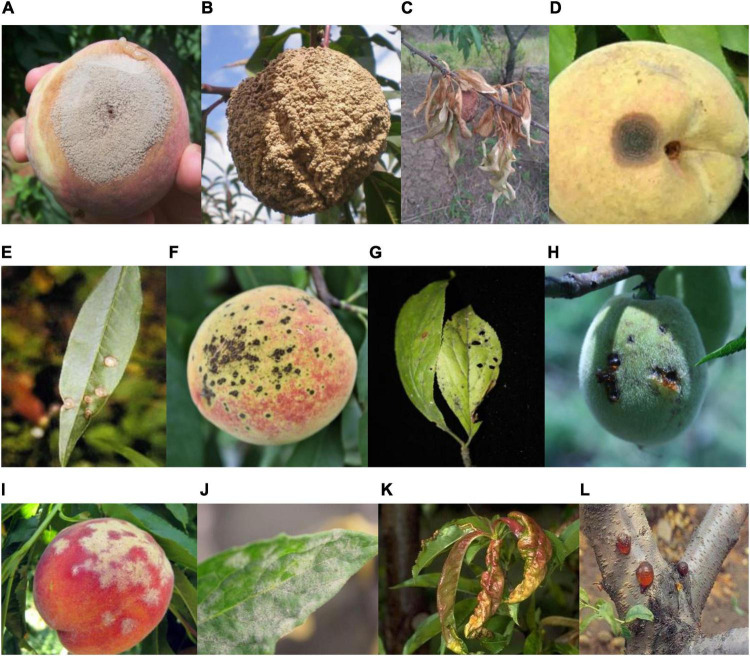
Major plant diseases of peach. **(A)** Brown rot of fruit, **(B)** brown rot of fruit, **(C)** brown rot of leaf, **(D)** anthracnose of fruit, **(E)** anthracnose of leaf, **(F)** scab of fruit, **(G)** scab of leaf, **(H)** bacterial shot hole of fruit, **(I)** powdery mildew of fruit, **(J)** powdery mildew of leaf, **(K)** leaf curl of a leaf, and **(L)** gummosis of a branch (Yao et al., 2021).

The original images of peach diseases (see Figure 1, Yao et al., 2021 for detail) were collected to form the Peach Disease Image Dataset (PDID). The numbers of images acquired for brown rot disease, anthracnose disease, scab disease, bacterial shot hole disease, gummosis disease, powdery mildew disease, and leaf curl disease were 94, 157, 654, 427, 91, 50, and 87, respectively (see Table 1 for detail). As can be seen, the distribution of the number of images in PDID is imbalanced.

**TABLE 1:** Classification of peach disease image dataset.

Class	Part	Sample	Class	Part	Sample
Brown rot	Fruits	88	Bacterial shot hole	Fruits	193
	Leaves	6		Leaves	229
Anthracnose	Fruits	129		Branches	5
	Leaves	28	Gummosis	Branches	91
Scab	Fruits	614	Powdery mildew	Fruits	32
	Leaves	35		Leaves	18
	Branches	5	Leaf curl	Leaves	87

Figure 1 Seven categories of disease images.

In order to distinguish the severity of each disease in more detail, we divided each disease into three levels: early disease, middle disease, and end disease. After division, the number of classes changed from 7 to 21, which are as follows: early brown rot, middle brown rot, end brown rot, early anthracnose, middle anthracnose, end anthracnose, early scab, middle scab, end scab, early gummosis, middle gummosis, end gummosis, early leaf curl, middle leaf curl, end leaf curl, early bacterial shot hole, middle bacterial shot hole, and end bacterial shot hole. However, the number of images per class is still small. To increase the number of images, we performed data augmentation (flipping, rotation, adding noise, and changing saturation) on the images. Finally, the number of samples included in the study was 5,627. These samples were divided into 4,051 training samples, 1,013 validation samples, and 563 testing samples.

**FIGURE 2:**
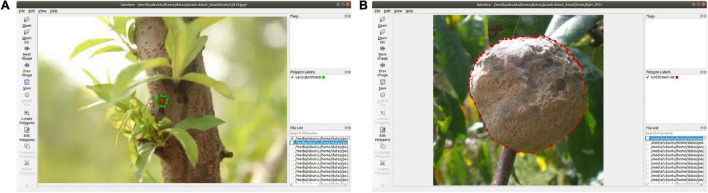
Marking process: **(A)** early gummosis and **(B)** end brown rot.

Labelme software was used to mark the lesion area in the images of different peach diseases. Figure 2 shows the marking process of early gummosis and end brown rot. After a picture is marked, it is saved as a json file, and the key points and disease names are included in the json file. Mask R-CNN and Mask Scoring R-CNN use the same dataset format, and convert the saved json file to COCO dataset format.

### Mask R-CNN

Mask R-CNN and Mask Scoring R-CNN are representatives of typical instance segmentation tasks, and Mask Scoring R-CNN is the improved version of Mask R-CNN. In order to obtain more effective information about the peach disease, two instance segmentation networks (Mask R-CNN and Mask Scoring R-CNN) with focal loss are used to segment peach diseases. As Mask Scoring R-CNN is based on Mask R-CNN, this paper used focal loss in Mask R-CNN and Mask Scoring R-CNN separately.

**FIGURE 3:**
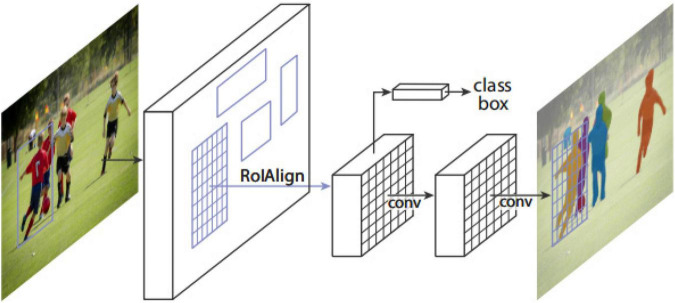
The Mask R-CNN framework for instance segmentation (He et al., 2017).

The Mask R-CNN framework for instance segmentation task is shown in Figure 3 (He et al., 2017). Mask R-CNN adopts a two-stage procedure. The first stage is RPN. In the second stage, in parallel to predicting the class and box offset, Mask R-CNN also outputs a binary mask for each RoI. Mask R-CNN follows the spirit of Fast R-CNN that applies bounding box classification and regression in parallel. Formally, during training, Mask R-CNN defines a multi-task loss on each sampled RoI as *L* = *L*_*cls*_ + *L*_*box*_ + *L*_*mask*_. The classification loss *L*_*cls*_and bounding box loss *L*_*box*_are the same as those defined by a previous study (Girshick, 2015). The mask branch has a *Km*^2^-dimensional output for each RoI, which encodes K binary masks of resolution*m*×*m*, one for each of the K classes. It applies a per-pixel sigmoid and defines *L*_*mask*_as the average binary cross-entropy loss. For an RoI associated with ground-truth class k, *L*_*mask*_ is only defined on the k-th mask. The definition of *L*_*mask*_ allows the network to generate masks for every class without competition among the classes. The dedicated classification branch is relied upon to predict the class label used to select the output mask, which decouples mask and class prediction.

### Mask Scoring R-CNN

**FIGURE 4:**
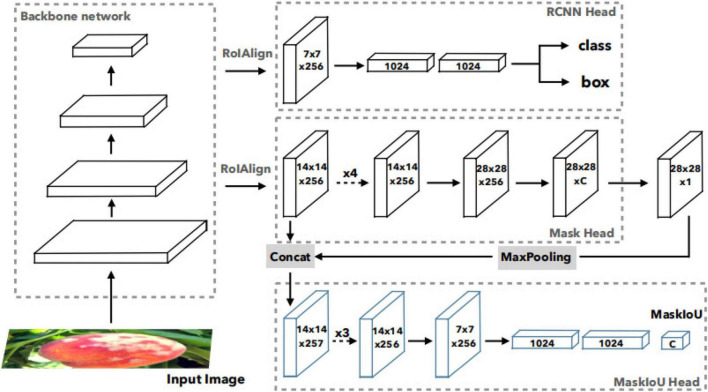
Network architecture of Mask Scoring R-CNN (Huang et al., 2019).

In Mask R-CNN framework, the score of instance segmentation hypothesis is determined by the largest element in its classification scores, which can be obtained in R-CNN. But classification score and ground truth mask are not well correlated in Mask R-CNN. So, Mask Scoring R-CNN was proposed. Figure 4 (Huang et al., 2019) shows the network architecture of Mask Scoring R-CNN, which is a Mask R-CNN with an additional MaskIoU head module that learns the MaskIoU aligned mask score. The input image is fed into a backbone network to generate RoIs *via* RPN and RoI features *via* RoIAlign. The R-CNN head and Mask head are standard components of Mask R-CNN. For predicting MaskIoU, the predicted mask and RoI feature are used as input. The MaskIoU head has four convolution layers (all have kernel = 3 and the final one uses stride = 2 for downsampling) and three fully connected layers (the final one outputs C classes MaskIoU.). During inference, the predicted MaskIoU is multiplied by the classification score to get the new calibrated mask score as the final mask confidence. Mask Scoring R-CNN defines*S*_*mask*_ as the score of the predicted mask. The ideal *S*_*mask*_is equal to the pixel-level IoU between the predicted mask and its matched ground truth mask, which also should have only a positive value for the ground truth category and zero for other classes, since a mask belongs to one class only. This requires the mask score to work well on two tasks: classifying the mask to the right category and regressing the proposed MaskIoU for the foreground object category. So, *S*_*mask*_ = *S*_*cls*_∙*S*_*iou*_is denoted for all object categories. *S*_*cls*_focuses on classifying the proposal to the corresponding class, and *S*_*iou*_focuses on regressing the MaskIoU. A classification score can be obtained in the classification task in the R-CNN stage. The MaskIoU head aims to regress the IoU between the predicted mask and its ground truth mask. The predicted MaskIoU scores are multiplied with classification score to get the new calibrated mask score as the final mask confidence. The concatenation of features from the RoIAlign layer and the predicted mask is the input of MaskIoU head. When concatenating, it uses a max pooling layer with kernel size of 2 and stride of 2 to enable the predicted mask to have the same spatial size as the RoI feature. MaskIoU head consists of four convolution layers and three fully connected layers. For the four convolution layers, it follows Mask head and sets the kernel size and filter number to 3 and 256, respectively, for all the convolution layers. For the three fully connected (FC) layers, it follows the R-CNN head and set the outputs of the first two FC layers to 1,024 and the output of the final FC to the number of classes.

### Image Pre-processing

The samples in the dataset are RGB images. Generally, images were processed as follows: First, Z-Score normalization was performed. Precisely, mean value m_*x*_and standard deviation*s*_*x*_ were calculated. Then, for each pixel value*x*as input, input *x* is changed to*x*′ = *x*−*m*_*x*_/*s*_*x*_, so that the normalized data was a standard normal distribution with zero mean and unit variance. After that, several augmentations, including random flipping, resize, and Pad (size = 32), were used for training and validating the dataset. The augmentation was helpful for enhancing the generalization ability of the model and preventing overfitting.

### Improved Method

As the number of samples in the peach disease image dataset is relatively small and the samples in this dataset were imbalanced, standard machine learning techniques have low accuracy. To improve the segmentation accuracy, focal Loss was used for this dataset.

The focal loss is defined as follows:


(1) $$F\,L\,\left(p_{t}\right)=-\alpha_{t}\,{\left(1-p_{t}\right)}^{\gamma }\,\log\left(p_{t}\right)$$

where $p_{t}=\left\{\begin{array}{ll} p & \text{if}\;y=1\\ 1-p & \text{otherwise} \end{array}\right.$ and $\alpha_{t}=\left\{\begin{array}{ll} \alpha & \text{if}\;y=1\\ 1-\alpha & \text{otherwise} \end{array}\right.$, for binary classification, y∈{ ± 1} specifies the ground truth class, and p∈[0, 1] is the model’s estimated probability for the class with label *y* = 1. Weighting factor α ∈ [0, 1] for class 1 and 1-α for class-1. While αbalances the importance of positive/negative examples, it does not differentiate between easy/hard examples. This focal loss function gives smaller weights to easy examples. This helps the training method to focus on hard negatives. A modulating factor (1−*p*_*t*_)^γ^ is added to the cross-entropy loss, with tunable focusing parameter γ ≥ 0.

When an example is misclassified and *p*_*t*_is small, the modulating factor is near 1, and the loss is unaffected. When *p*_*t*_ is near 1, the factor (1−*p*_*t*_)^γ^ is close to 0, and the loss for well-classified examples is downweighted. The focusing parameter γsmoothly adjusts the rate at which easy examples are downweighted. When γ = 0, FL is equivalent to CE, and as γ is increased, the effect of the modulating factor is likewise increased. Intuitively, the modulating factor reduces the loss contribution from easy examples and extends the range in which an example receives low loss.

### Implementation

The experiment of classification was performed on a CentOS workstation equipped with two Intel(R) Xeon(R) E5-2683 v4 CPU (55G RAM) and accelerated by two Tesla P100-PCIE GPU (16 GB memory). The model implementation in this paper was powered by the deep learning framework of Pytorch.

## Results and Discussion

In this study, mAP (mean average precision) is used as an evaluation indicator, which is usually used in instance segmentation tasks. The experiments based on MMDetection and bbox_mAP_50 represent mAP of BBox when IoU is 0.5. Also, segm_mAP_50 represents mAP of segmentation when IoU is 0.5, while R50 represents ResNet50.

Using focal loss, the empirical values given in the current study (Dai et al., 2017) are γ = 2 andα = 0.25, but different data distributions require different parameters, so different gamma (γ) and alpha (α) values were tested, and the results are presented in Table 2. When γ = 2 and α = 0.95, the result is improved using Mask R-CNN. But when Mask Scoring R-CNN was used, γ = 4 and α = 0.45 provides better results, as given in Table 3. FL represents the focal loss in Tables 2, 3, and the learning rate is 0.00025 in all the experiments.

**TABLE 2:** Training parameter and test results based on Mask R-CNN with different loss functions.

Network	Bbox_mAP_50	Segm_mAP_50	Loss	Backbone	Epoch	γ	α
Mask R-CNN	0.396	0.236	CE	R50	12		
Mask R-CNN	0.416	0.224	FL	R50	12	5	0.95
Mask R-CNN	0.428	0.197	FL	R50	12	2	0.25
Mask R-CNN	0.463	0.219	FL	R50	12	2	0.55
Mask R-CNN	0.515	0.236	FL	R50	12	2	0.75
Mask R-CNN	0.540	0.246	FL	R50	12	2	0.85
Mask R-CNN	0.534	0.254	FL	R50	12	2	0.95
Mask R-CNN	0.518	0.219	FL	R50	12	1	0.95
Mask R-CNN	0.465	0.222	FL	R50	12	3	0.95
Mask R-CNN	0.443	0.215	FL	R50	12	4	0.95

**TABLE 3:** Training parameter and test results based on Mask Scoring R-CNN with different loss functions.

Network	Bbox_mAP_50	Segm_mAP_50	Loss	Backbone	Epoch	γ	α
Mask Scoring R-CNN	0.367	0.246	CE	R50	12		
Mask Scoring R-CNN	0.367	0.224	FL	R50	12	5	0.95
Mask Scoring R-CNN	0.425	0.243	FL	R50	12	5	0.75
Mask Scoring R-CNN	0.451	0.251	FL	R50	12	5	0.55
Mask Scoring R-CNN	0.425	0.240	FL	R50	12	5	0.25
Mask Scoring R-CNN	0.472	0.274	FL	R50	12	4	0.45
Mask Scoring R-CNN	0.408	0.238	FL	R50	12	3	0.35
Mask Scoring R-CNN	0.346	0.196	FL	R50	12	1	0.05
Mask Scoring R-CNN	0.450	0.259	FL	R50	12	2	0.25

**FIGURE 5:**
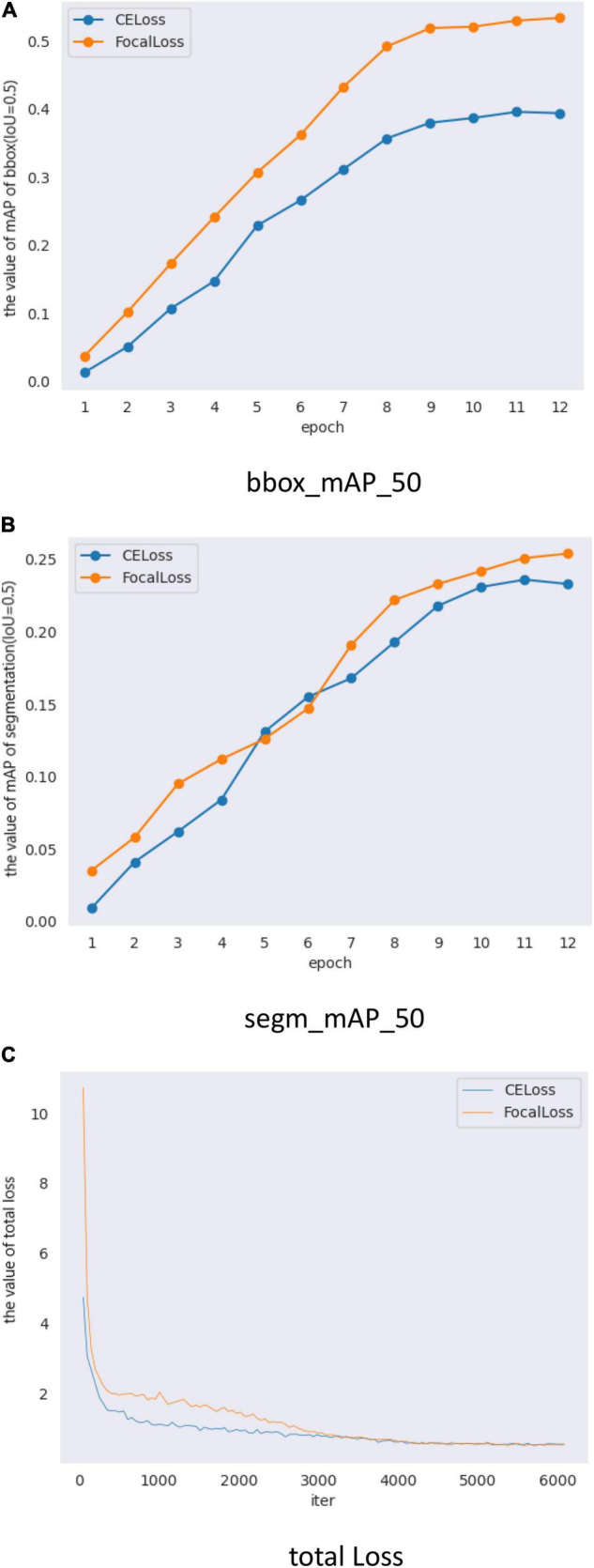
Mask R-CNN with different loss validation parameters and loss functions. **(A)** Comparison of mAP_50 of bbox (IoU = 0.5) on different loss, **(B)** Comparison of mAP_50 of segmentation (IoU = 0.5) on different loss, and **(C)** Comparison of total loss.

In all the experiments of this study, the following parameters are similar: neck using FPN, loss_BBox of Rpn-head using L1 Loss, loss-cls of BBox-head using CE, and loss-BBox of BBox-head using L1 loss in roi_head and loss-mask of mask-head using CE. The focal loss was used in RPN. When focal loss and CE loss were used in RPN, the obtained BBox_mAP_50 and segm_mAP_50 metrics are presented in Table 4. The test results shows BBox_mAP_50 increased from 0.396 to 0.534 and segm_mAP_50 increased from 0.236 to 0.254 (Table 4). Mask R-CNN with a different loss function used the same training parameters (epoch, learning rate, and batch size). Figure 5A shows the validation mAP of BBox (IoU = 0.5) from 1 to 12 epochs when training the dataset with different loss functions, displaying that the validation mAP of BBox is higher with focal loss than with CE loss. Figure 5B shows the validation mAP of segmentation (IoU = 0.5) from 1 to 12 epochs when training the dataset with different loss functions, displaying that the validation mAP of segmentation is higher with focal loss than with CE loss. However, the increment in the map of segmentation is lower than BBox. Figure 5C shows the total loss value of the *y*-axis changes with the changed iter value of the *x*-axis when training the dataset with different loss functions. The results presented in Figure 5 and Table 4 show that the application of Mask R-CNN with focal loss achieves better performance compared with CE loss.

**TABLE 4:** Training parameter and test results based on Mask R-CNN with different loss functions.

Network	Bbox_ mAP_50	Segm_ mAP_50	Loss	Backbone	Epoch	γ	α
Mask R-CNN	0.396	0.236	CE	R50	12		
Mask R-CNN	0.534	0.254	FL	R50	12	2	0.95

**FIGURE 6:**
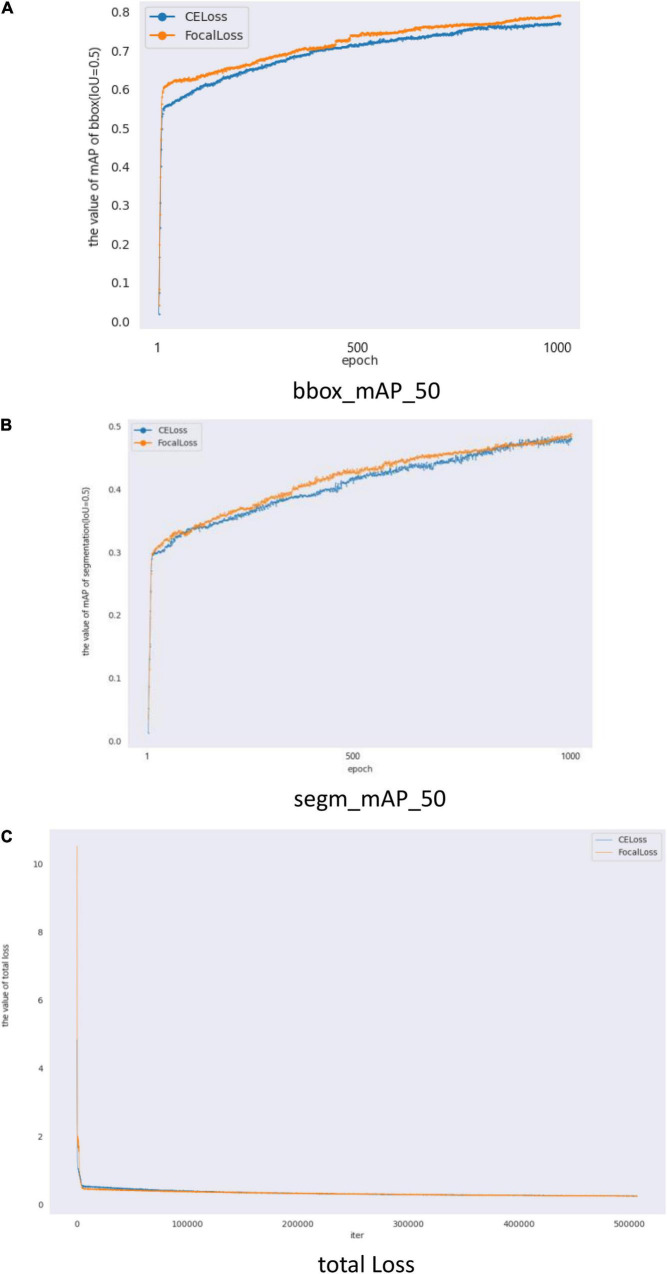
Mask R-CNN with different loss validation parameters and loss functions. **(A)** Comparison of mAP_50 of bbox (IoU = 0.5) on different loss, **(B)** Comparison of mAP_50 of segmentation (IoU = 0.5) on different loss, and **(C)** Comparison of total loss.

In general, deeper networks and larger epochs can provide better results. When epoch (1,000) and backbone (ResNetx101) are changed, the results obtained are displayed in Figure 6 and Table 5. Figure 6A shows the validation mAP of BBox (IoU = 0.5) from 1 to 1,000 epochs when training the dataset with different loss functions, displaying validation mAP of BBox is higher with focal loss than with CE loss. Figure 6B shows validation mAP of segmentation (IoU = 0.5) from 1 to 1,000 epochs when training the dataset with different loss functions, displaying validation mAP of segmentation is higher with focal loss than with CE loss. Figure 6C shows the total loss value of the *y*-axis changes with the changed iter value of the *x*-axis when training the dataset with different loss functions. The results presented in Figure 6 and Table 5 also show that the application of Mask R-CNN with focal loss achieves better performance compared to CE loss. Rx101 represents ResNetx101 in Table 5. The test results in Table 5 show that BBox_mAP_50 increased from 0.749 to 0.771 and segm_mAP_50 increased from 0.452 to 0.463. It can be seen that with the increase of epochs and the deepening of network depth, a better effect is achieved. Despite changing epoch and backbone, the results presented in Figure 6 and Table 5 show that Mask R-CNN with focal loss achieves better performance compared to CE loss.

**TABLE 5:** Training parameter and test results based on Mask R-CNN with different loss functions.

Network	Bbox_ mAP_50	Segm_ mAP_50	Loss	Backbone	Epoch	γ	α
Mask R-CNN	0.749	0.452	CE	Rx101	1000		
Mask R-CNN	0.771	0.463	FL	Rx101	1000	2	0.95

**FIGURE 7:**
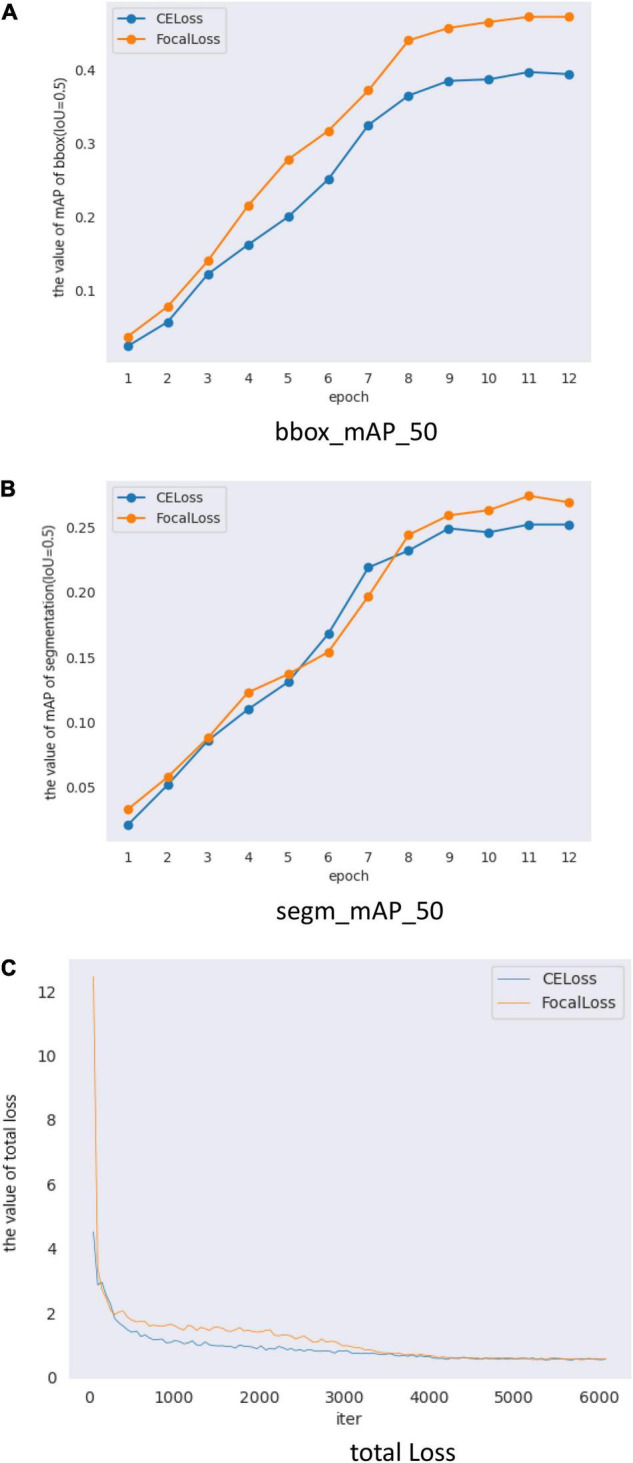
Mask Scoring R-CNN with different loss validation parameters and loss functions. **(A)** Comparison of mAP_50 of bbox (IoU = 0.5) on different loss, **(B)** Comparison of mAP_50 of segmentation (IoU = 0.5) on different loss, and **(C)** Comparison of total loss.

Figure 7 and Table 6 show the results of Mask Scoring R-CNN. When focal loss and CE loss are used in RPN, the obtained BBox_mAP_50 and segm_mAP_50 metrics are presented in Table 6. The test results show BBox_mAP_50 increased from 0.387 to 0.472 and segm_mAP_50 increased from 0.252 to 0.274 (Table 6). Figure 7A shows the validation mAP of BBox (IoU = 0.5) from 1 to 12 epochs when training the dataset with different loss functions, displaying that the validation mAP of BBox is higher with focal loss than with CE loss. Figure 7B shows validation mAP of segmentation (IoU = 0.5) from 1 to 12 epochs when training the dataset with different loss functions, displaying that validation mAP of segmentation is higher with focal loss than with CE loss. Figure 7C shows the total loss value of the *y*-axis changes with the changed iter value of the *x*-axis when training the dataset with different loss functions. The results presented in Figure 7 and Table 6 show that the application of Mask Scoring R-CNN with focal loss achieves better performance compared to CE loss. Comparing the data presented in Tables 4, 6, it can be found that the mAP of segmentation based on Mask Scoring R-CNN is higher than that based on Mask R-CNN and also that the focal loss produces effective results.

**TABLE 6:** Training parameter and test results based on Mask Scoring R-CNN with different loss functions.

Network	Bbox_ mAP_50	Segm_ mAP_50	Loss	Backbone	Epoch	γ	α
Mask Scoring R-CNN	0.387	0.252	CE	R50	12		
Mask Scoring R-CNN	0.472	0.274	FL	R50	12	4	0.45

**FIGURE 8:**
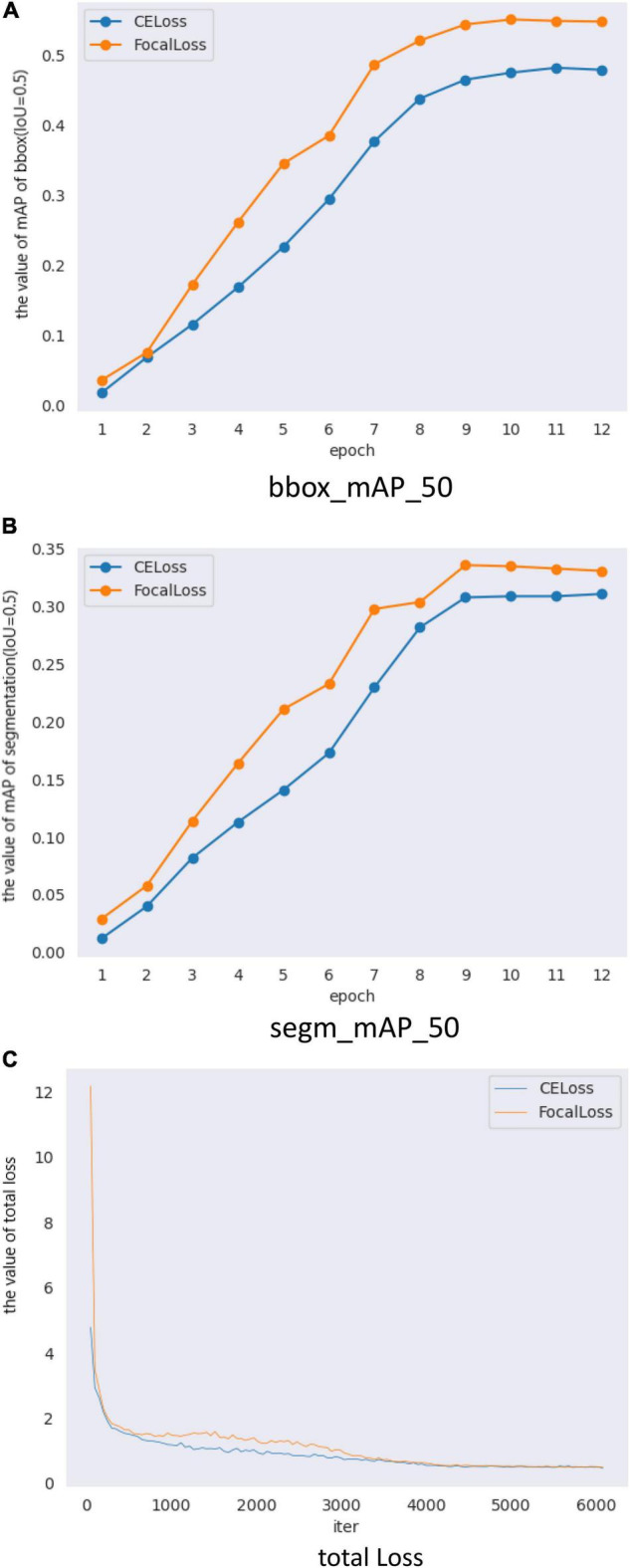
Mask Scoring R-CNN with different loss validation parameters and loss functions. **(A)** Comparison of mAP_50 of bbox (IoU = 0.5) on different loss, **(B)** Comparison of mAP_50 of segmentation (IoU = 0.5) on different loss, and **(C)** Comparison of total loss.

When we only changed the backbone from ResNet50 to ResNetx101, the results of training and testing are shown in Figure 8 and Table 7. The test results in Table 7 show that BBox_mAP_50 increased from 0.479 to 0.544 and segm_mAP_50 increased from 0.311 to 0.336. It can be seen that an increase in network depth can improve object detection and segmentation effect. Although the backbone was changed, the results presented in Figure 8 and Table 7 show that Mask Scoring R-CNN with focal loss achieves better performance compared to CE loss.

**TABLE 7:** Training parameter and test results based on Mask Scoring R-CNN with different loss functions.

Network	Bbox_ mAP_50	Segm_ mAP_50	Loss	Backbone	Epoch	γ	α
Mask Scoring R-CNN	0.479	0.311	CE	Rx101	12		
Mask Scoring R-CNN	0.544	0.336	FL	Rx101	12	4	0.45

Table 8 shows the results of Mask R-CNN/Mask Scoring R-CNN with focal loss compared to other methods. Cascade-DCN represents Cascade R-CNN with deformable convolutional networks. Mask-DCN represents Mask R-CNN with deformable convolutional networks. Mask-DCNV2 represents Mask R-CNN with deformable convolutional networks V2. Other hyperparameters of these six methods are similar. The last HRNet used the same learning rate and epoch, but the backbone was different. Using focal loss with Mask R-CNN and Mask Scoring R-CNN provides better segmentation results compared to other methods.

**TABLE 8:** Results of the method proposed in this study compared to other methods.

Network	Bbox_ mAP_50	Segm_ mAP_50	Loss	Backbone	Epoch	γ	α
Mask R-CNN	0.534	0.254	FL	R50	12	2	0.95
Mask Scoring R-CNN	0.472	0.274	FL	R50	12	4	0.45
Cascade R-CNN	0.450	0.243	CE	R50	12		
Cascade-DCN	0.447	0.250	CE	R50	12		
Mask-DCN	0.397	0.222	CE	R50	12		
Mask-DCNV2	0.232	0.127	CE	R50	12		
HRNet	0.303	0.183	CE	HRNet	12		

**FIGURE 9:**
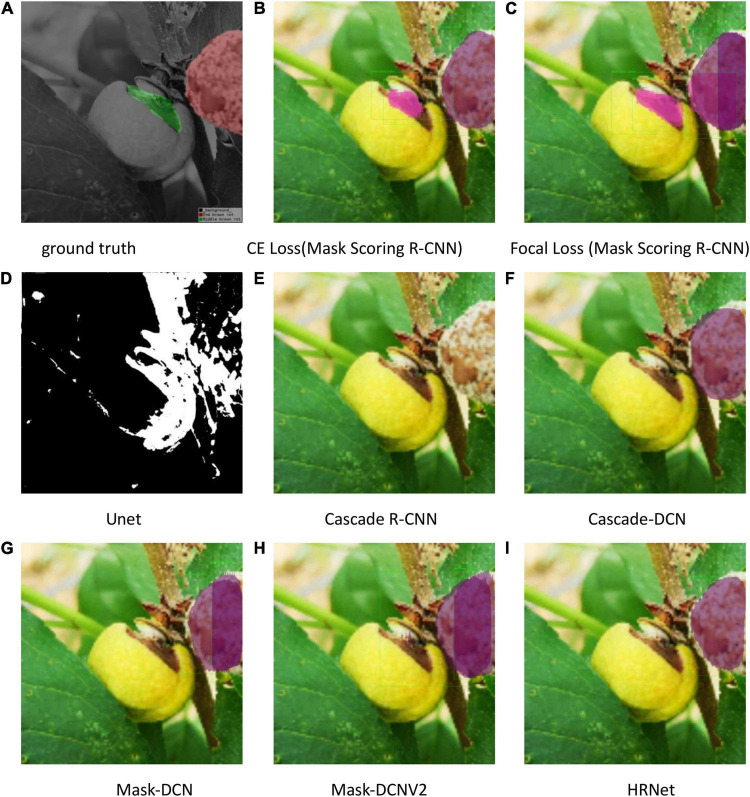
Test results of different methods. **(A)** Ground truth, **(B)** CE Loss on Mask Scoring R-CNN, **(C)** Focal Loss on Mask Scoring R-CNN, **(D)** Unet, **(E)** Cascade R-CNN, **(F)** Cascade-DCN, **(G)** Mask-DCN, **(H)** Mask-DCNV2, and **(I)** HRNet.

**FIGURE 10:**
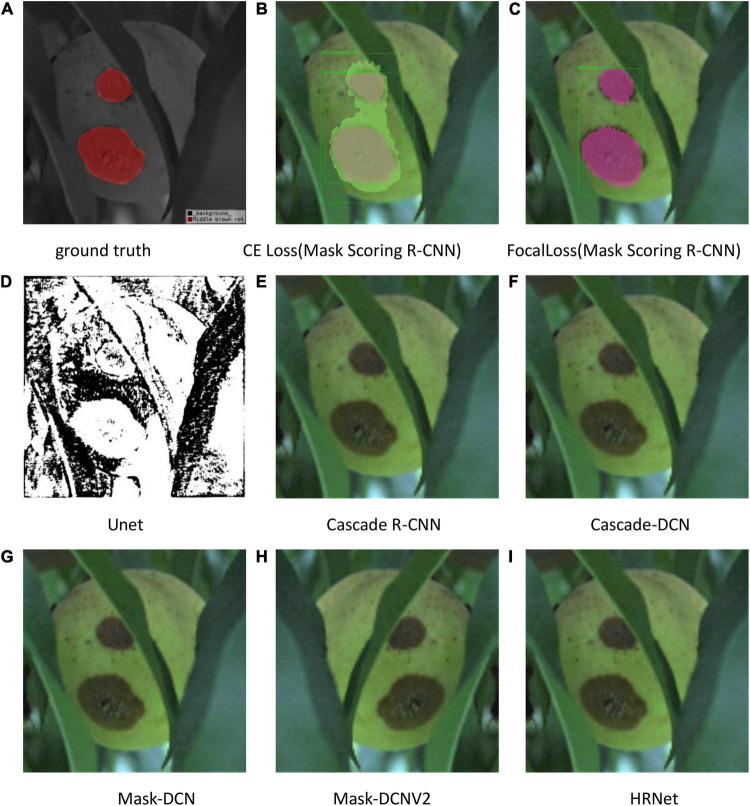
Test results of different methods. **(A)** Ground truth, **(B)** CE Loss on Mask Scoring R-CNN, **(C)** Focal Loss on Mask Scoring R-CNN, **(D)** Unet, **(E)** Cascade R-CNN, **(F)** Cascade-DCN, **(G)** Mask-DCN, **(H)** Mask-DCNV2, and **(I)** HRNet.

**FIGURE 11:**
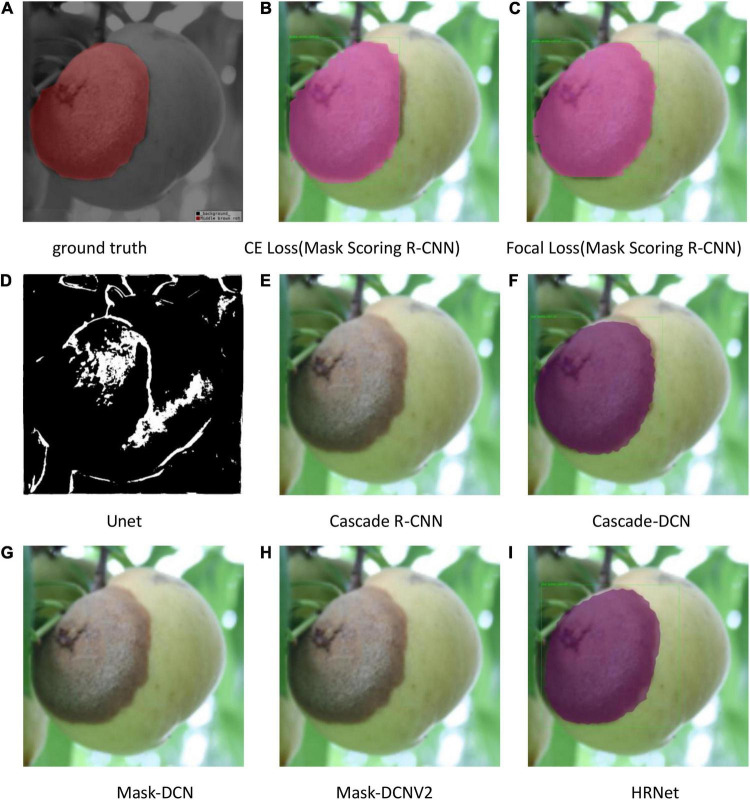
Test results of different methods. **(A)** Ground truth, **(B)** CE Loss on Mask Scoring R-CNN, **(C)** Focal Loss on Mask Scoring R-CNN, **(D)** Unet, **(E)** Cascade R-CNN, **(F)** Cascade-DCN, **(G)** Mask-DCN, **(H)** Mask-DCNV2, and **(I)** HRNet.

The test results are shown in Figures 9–11. All peach diseases were tested. Mask Scoring R-CNN with focal loss consistently produced better segmentation results: (1) Mask Scoring R-CNN with CE loss provides a segmentation that cannot cover some ground truth regions. Mask Scoring R-CNN with focal loss provides more correct segmentation results for the diseases brown rot, gummosis, leaf curl, and anthracnose. The test results for the brown rot disease are shown in Figures 9, 11. (2) Mask Scoring R-CNN with CE loss produces a segmentation that covers regions not in the ground truth. Mask Scoring R-CNN with focal loss gives fewer false segmentations for the diseases like bacterial shot hole and brown rot. The test result for the disease brown rot is shown in Figure 10. (3) Mask Scoring

R-CNN with CE loss presents no detection and segmentation, while Mask Scoring R-CNN with focal loss provides detection and segmentation. The data presented in Figures 9D–11D are tested by the U-Net model, which illustrates that the results are poor. Since the three test images have complex backgrounds, the lesion areas were not segmented well from the background. However, when the lesion areas and background are relatively simple, the segmentation is better.

The results obtained by conducting the same experiments on the original dataset are summarized in Table 9. The original dataset (PDID) includes seven peach diseases, with 1,560 images. The ratio of training samples, validation samples, and test samples is 7:2:1. Table 9 shows that focal loss can improve the mAP of BBox and segmentation on both Mask R-CNN and Mask Scoring R-CNN tasks. But, the parameters of γandαneed to get the optimal value through experiments. The parameters will be different when the dataset is different.

**TABLE 9:** Training parameters and test results on original dataset.

Network	Bbox_ mAP_50	Segm_ mAP_50	Loss	Backbone	Epoch	γ	α
Mask R-CNN	0.280	0.260	CE	R50	12		
Mask R-CNN	0.294	0.267	FL	R50	12	5	0.95
Mask Scoring R-CNN	0.293	0.264	CE	R50	12		
Mask Scoring R-CNN	0.301	0.279	FL	R50	12	5	0.75
Mask Scoring R-CNN	0.333	0.313	CE	Rx101	24		
Mask Scoring R-CNN	0.371	0.333	FL	Rx101	24	5	0.75

## Conclusion

In this study, the output of this method provides information regarding the names of peach disease, disease severity levels, and masked lesion areas. Hence, detailed information about the diseases, not limited to disease names, can be obtained. Usually, disease names can be obtained by classification tasks. Data pertaining to disease names, disease severity level, and masked lesion areas are usually achieved by instance segmentation tasks. This study used the focal loss to improve the effect of instance segmentation. Due to the difficulty in obtaining the pictures of peach disease, the peach disease dataset often has unbalanced or hard samples. We used focal loss in the first stage, and segmentation results were found to be improved. Focal loss was used in Mask R-CNN and Mask Scoring R-CNN for classification, location, and segmentation of peach diseases, while getting better segmentation results. When using Mask R-CNN with ResNet50 as a backbone network, the focal loss parameters gamma (γ) was 2.0 and alpha (α) was 0.95. When Mask Scoring R-CNN was used with ResNet50 and ResNetx101 as the backbone network, the focal loss parameters gamma was 4.0 and alpha was 0.45. We also observed that the deeper the backbone network, the better the effect of focal loss. When dataset is changed, the parameters of γandαare different. Additionally, the U-Net model was used to segment the lesion areas of peach disease images, but the results showed that this model has a poor accuracy in complex background images. So, the method adopted in this study can improve the segmentation results and can also provide the disease names and severity (early, middle, and end), by displaying the lesion areas by mask. Thus, this technique can provide more detailed information for effective disease treatment and analysis.

## Data Availability Statement

The raw data supporting the conclusions of this article will be made available by the authors, without undue reservation.

## Author Contributions

FN devised the study in collaboration with W-KS, GL, and HW. MW carried out experimental work partly. All authors read and approved the manuscript.

## Conflict of Interest

The authors declare that the research was conducted in the absence of any commercial or financial relationships that could be construed as a potential conflict of interest.

## Publisher’s Note

All claims expressed in this article are solely those of the authors and do not necessarily represent those of their affiliated organizations, or those of the publisher, the editors and the reviewers. Any product that may be evaluated in this article, or claim that may be made by its manufacturer, is not guaranteed or endorsed by the publisher.
